# Decompressive craniectomy in the management of intracranial hypertension after traumatic brain injury: a systematic review and meta-analysis

**DOI:** 10.1038/s41598-017-08959-y

**Published:** 2017-08-18

**Authors:** Danfeng Zhang, Qiang Xue, Jigang Chen, Yan Dong, Lijun Hou, Ying Jiang, Junyu Wang

**Affiliations:** Department of Neurosurgery, Shanghai neurosurgical institute, Changzheng Hospital, Shanghai, China

## Abstract

We aim to perform a systematic review and meta-analysis to examine the prognostic value of decompressive craniectomy (DC) in patients with traumatic intracranial hypertension. PubMed, EMBASE, Cochrane Controlled Trials Register, Web of Science, http://clinicaltrials.gov/ were searched for eligible studies. Ten studies were included in the systematic review, with four randomized controlled trials involved in the meta-analysis, where compared with medical therapies, DC could significantly reduce mortality rate [risk ratio (RR), 0.59; 95% confidence interval (CI), 0.47–0.74, *P* < 0.001], lower intracranial pressure (ICP) [mean difference (MD), −2.12 mmHg; 95% CI, −2.81 to −1.43, *P* < 0.001], decrease the length of ICU stay (MD, −4.63 days; 95% CI, −6.62 to −2.65, *P* < 0.001) and hospital stay (MD, −14.39 days; 95% CI, −26.00 to −2.78, *P* = 0.02), but increase complications rate (RR, 1.94; 95% CI, 1.31–2.87, *P* < 0.001). No significant difference was detected for Glasgow Outcome Scale at six months (RR, 0.85; 95% CI, 0.61–1.18, *P* = 0.33), while in subgroup analysis, early DC would possibly result in improved prognosis (*P* = 0.04). Results from observational studies supported pooled results except prolonged length of ICU and hospital stay. Conclusively, DC seemed to effectively lower ICP, reduce mortality rate but increase complications rate, while its benefit on functional outcomes was not statistically significant.

## Introduction

Traumatic brain injury (TBI) is a major health problem usually complicated with intracerebral hemorrhage, brain swelling and hydrocephalus and eventually leads to elevated intracranial pressure (ICP)^1–3^. As demonstrated in most studies, intracranial hypertension (ICH) is correlated to the increased incidence of death and severe disability following TBI^4^. Thus, monitoring and reversing of ICP are essential in the management of TBI and routinely used in some trauma centers^5^. Though medical treatments including hyperosmolar therapy, sedation, barbiturate coma, therapeutic hypothermia and ventricular drainage prove to be effective, there do exist a set of patients resistant to these treatment modalities when brain swelling continues, and finally resulting in refractory ICH (RICH)^6, 7^.

Decompressive craniectomy (DC) is a surgical procedure that has regained much interests in the management of RICH after TBI in recent years^8^. DC can be categorized to be primary and secondary. Primary DC is often performed in acute phase after TBI and refers to the surgery leaving a large bone flap out after evacuation of intracranial lesions^9^. Secondary DC is often conducted as the last resort for malignant elevation of ICP when medical therapies failed, so early trials taking DC as a premature choice were frustrating with patients in DC group showing high mortality and unfavorable functional outcomes^10^. But recently some studies, including a large scale randomized controlled trial (RCTs, RESCUEicp trial), found that DC could reduce ICP and mortality, improve prognosis in comparison with medical therapies^11^. However, the effects of secondary DC are still controversial and worth further exploring^12^.

The present systematic review and meta-analysis aims to comprehensively summarize and quantify the effects of DC interventions on overall mortality rate and ICP as well as long-term prognosis in TBI patients.

## Results

### Literature Search

**Figure 1:**
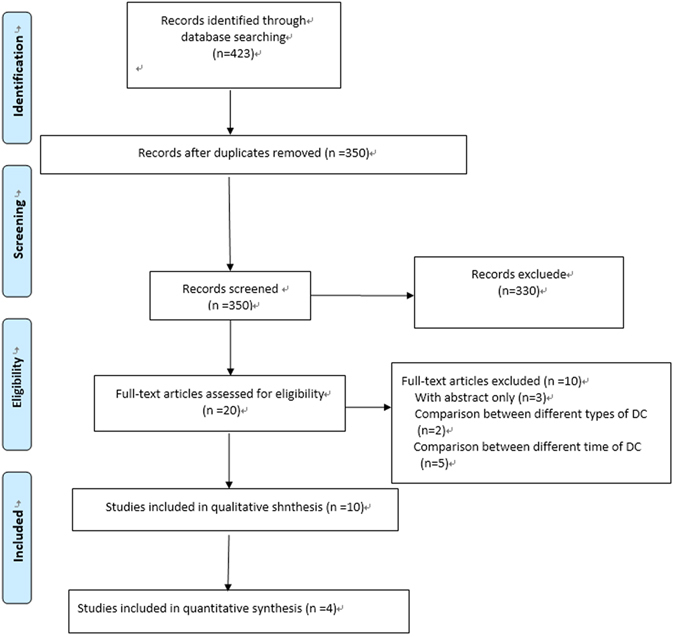
Flow diagram of study selection.

A total of 423 studies were retrieved from the initial search, among which 20 were potentially related to our review and the full texts were reviewed. Of these 20 studies, 10 were excluded for various reasons, which were shown in Fig. 1. Therefore, a total of 10 eligible studies were included in our systematic review, with four RCTs in the meta-analysis.

### Study Characteristics

Main characteristics of the 10 studies were shown in Table 1. There were four RCTs^6, 11, 13, 14^, five retrospective studies^9, 15–18^ and one prospective study^19^, totaling 1390 patients in the systematic review and 654 in meta-analysis (325 DCs, 329 non-DCs). Patients’ age ranged from seven to 40.2 years, and most of the participants were male. The mean baseline Glasgow Coma Scale (GCS) score of participants ranged from three to 6.9.

**Table 1:** Characteristics of included studies.

First author (year)	Study design	Patients	Time interval to treatment	Outcome assessments	Treatment	*N* of patients	Detailed description	Age, Men (%)	Baseline characteristics (GCS at Baseline)
Taylor^14^	Randomized trial	Children over 12 months, sustained a TBI and ICH or had evidence of herniation.	Median: 19.2 (range: 7.3–29.3) hours after injury	ICP, CPP, duration of stay, GOS	DC	13	A bitemporal DC via a bilateral vertical incision in the mid-temporal region and medical management	NA	Median: 6 (range 3–11)
					Medical therapy	14	Medical management alone	NA	Median: 5 (range 4–9)
Josan^16^	Retrospective study	Children with RICH after isolated severe TBI	NA	ICP, GOS	DC	6	A large frontotemporoparietal flap and leaving the dura intact without any attempt at duraplasty.	13, 5 (83.3)	6.83 ± 3.25
					Medical therapy	6	Non-operative treatment	11.5, 3 (50)	6 ± 2.28
Olivecrona^15^	Retrospective study	Severe TBI	Mean: 45 (range: 2–157) hours after treatment	GOS	DC	21	Unilaterally or bilaterally craniectomy based on the CT scan results	39.1, 15 (71.4)	Mean: 6.5 (range 3–8)
					Medical therapy	72	Patients were sedated with midazolam and fentanyl, or underwent ventriculostomy.	37.1, 56 (77.8)	Mean: 5.9 (range 3–8)
Rubiano^18^	Case control study	Age younger than 50 years with severe TBI	Within 12 hours from injury	LO-ICU, LOH, discharge status and GOS	DC	16	A decompressive fronto-temporo-parietal craniectomy, uni- or bilaterally according to the CT findings	18.3, 7 (43.8)	Mean: 4.5
					Medical therapy	20	NA	24.3, 14 (70)	Mean: 4.4
Qiu^6^	Randomized trial	Patients of unilateral acute posttraumatic brain swelling with midline shifting more than 5 mm	NA	ICP, GOS, the mortality rate and the complications	DC	37	Unilateral DC at the frontoparietotemporal region, based on the lesion location and midline shift determined by CT scans.	39.9, 27 (73.0)	Score:3–5 (24.3%); Score:6–8 (75.7%)
					Medical therapy	37	Unilateral routine temporoparietal craniectomy	40.2, 24 (64.9)	Score:3–5 (27%); Score:6–8 (73%)
Soustiel^19^	Prospective study	Patients more than 16 with severe TBI	Immediately after diagnostic tests and resuscitation measures.	CBF and metabolic rates, GOS	DC	36	Removal of a large frontal parietal temporal bone flap, Unilateral or bilateral decompression was based on CT scans	35.1,NA	5.8 ± 2.7
					Medical therapy	86	Mechanical ventilation, sedation induced by continuous infusion of propofol and fentanyl, and muscle relaxants as clinically required for ventilation purposes and ICP control	40.1, NA	6.5 ± 2.8
Thomale^17^	Retrospective study	Pediatric patients (≤16 years) with severe TBI	3 ± 3.98 (median: 2; range: 0–3.75) days post-trauma	Discharge of the ICU, ICP, GOS	DC	14	Bilateral fronto-temporo-parietal craniectomy, the dura mater was opened and a duraplasty performed	12, 8 (57.1)	Median: 6.5 (IQR 5–11)
					Medical therapy	39	Management according to a standardized protocol, first-line ICP treatment	7, 34 (87.2)	Median: 3 (IQR 3–6)
Cooper^13^	Randomized trial	Patients aged from 15 to 59 years and had a severe, nonpenetrating TBI	Within 72 hours after injury	Unfavorable outcome, GOS, ICP, ICP index, LO-ICU, LOH, and mortality	DC	73	A large bifrontotemporoparietal craniectomy with bilateral dural opening to maximize the reduction in ICP	23.7, 59 (81)	Median: 5 (IQR 3–7)
					Medical therapy	82	Standard care based on those recommended by the Brain Trauma Foundation included mild hypothermia (to 35 °C), the optimized use of barbiturates, or both	24.6, 61 (74)	Median: 6 (IQR 4–7)
Nirula^9^	Case control study	Patients aged more than 16 with blunt TBI	Within 48 hours after injury	Mortality, LOH, LO-ICU, complications	DC	210	DC was performed for relieving ICH or evacuating a space-occupying lesion within 48 hours of injury	40, 163 (77.6)	6.8 ± 3.0
					Medical therapy	210	Medical management	39, 167 (79.5)	6.9 ± 3.3
Hutchinson^11^	Randomized trial	Patients 10 to 65 years of age, with TBI and RICH (>25 mm Hg)	Within 4 to 6 hours after randomization	GOS, mortality, quality of life, LOH, GCS, ICP, economic evaluation.	DC	202	DC with medical therapy, either large unilateral frontotemporoparietal craniectomy or bifrontal craniectomy	32.3, 165 (81.7)	Score:1–2: 96 (53); Score:3–6: 85 (47)
					Medical therapy	196	Receiving continued medical therapy with the option of adding barbiturates	34.8, 156 (80)	Score:1–2: 85 (50); Score:3–6: 85 (50)

CBF, Cerebral Blood Flow; CPP, Cerebral Perfusion Pressure; CT, Computed Tomography; DC, Decompressive Craniectomy; ICH, Intracranial Hypertension; ICP, Intracranial Pressure; ICU: intensive care unit; IQR, Interquartile Range; GCS, Glasgow Coma Scale; GOS, Glasgow Outcome Scale; LOH, Length of Hospitalization; LO-ICU, Length of ICU Stay; NA, Not Available; RICH, Refractory Intracranial Hypertension; TBI, Traumatic Brain Injury.

### Quality Assessment

Risk of bias for each trial were assessed with the Cochrane risk of bias tool. (Supplementary Figures 1 and 2) All RCTs reported the randomization methods and allocation concealment in detail. Due to the nature of DC interventions, performing blinding methods to participants were usually impossible. So we assessed the performance bias according to the blinding of outcome assessors. In the domain of blinding of outcome assessment, three RCTs were at low risk of bias, while the other one was unclear due to the incomplete information on outcome assessment. For attrition bias, there were no dropouts or missing outcomes in three RCTs. But we found some missing outcome in one study. Additionally, protocols were available for two RCTs with one study’s primary outcome measure revised. We found no other suspect bias in four RCTs.

### Outcome Measures

DC-related outcomes are shown in Table 2.

**Table 2:** Outcomes of included studies.

First author (year)	Treatment	GOS Score at 3 Months	GOS Scores at 6 Months	GOS Scores at 12 Months	ICP level after Intervention (mm Hg)	Overall Mortality, n (%)	LOH (d)	LO-ICU (d)	N of patients with one or more complications
Taylor^14^	DC	NA	Favorable: 7 (53.8%); Unfavorable: 6 (46.2%)	NA	17.4 ± 3.4 (range: 11–25)	3 (23.1)	26.8 (range: 13.8–73.3)	9.6 (range: 1.7–31.2)	NA
	Medical therapy	NA	Favorable: 2 (14.3%); Unfavorable: 12 (85.7%)	NA	21.9 ± 8.5 (range: 11–44)	6 (42.9)	47.7 (range: 21.9–73.1)	12.8 (range: 1.0–14.8)	
Josan^16^	DC	NA	NA	Favorable: 6 (100%); Unfavorable: 0 (0)	12.33 ± 2.73	0	NA	NA	NA
	Medical therapy	NA	NA	Favorable: 3 (50%); Unfavorable: 3 (50%)	NA	2 (33.3)	NA	NA	
Olivecrona^15^	DC	NA	Favorable: 15 (71.4%); Unfavorable: 6 (28.6%)	NA	13.1 ± 2.1	NA	NA	NA	NA
	Medical therapy	NA	Favorable: 43 (60.6); Unfavorable: 28 (39.4)	NA	NA	NA	NA	NA	
Rubiano^18^	DC	NA	Favorable: 7 (44%); Unfavorable: 9 (56%)	NA	NA	4 (25)	23.4 (range: 5–57)	9.4 (range: 5–20)	NA
	Medical therapy	NA	Favorable: 0 (0%); Unfavorable: 20 (100%)	NA	NA	13 (65)	10.1 (range: 2–31)	5.9 (range: 2–13)	
Qiu^6^	DC	NA	Favorable: 21 (57%); Unfavorable: 16 (43%)	NA	24 h:15.19 ± 2.18; 48 h: 16.53 ± 1.53; 72 h: 15.98 ± 2.24; 96 h: 13.52 ± 2.33	10 (27)	NA	NA	NA
	Medical therapy	NA	Favorable: 12 (32%); Unfavorable: 25 (68%)	NA	24 h: 19.95 ± 2.24; 48 h: 18.32 ± 1.77; 72 h: 21.05 ± 2.23; 96 h: 17.68 ± 1.40	21 (57)	NA	NA	
Soustiel^19^	DC	NA	NA	NA	15.2 ± 12.5	NA	NA	16.1 ± 12.7	NA
	Medical therapy	NA	NA	NA	12.4 ± 8.7	NA	NA	19.5 ± 11.3	
Thomale^17^	DC	Median: 4 IQR(2.5–4.5)	NA	Median: 4 (IQR: 3, 5)	9.4 (range: 5.9–18.7)	NA	NA	Median: 20 (IQR: 4, 28.5)	NA
	Medical therapy	Median: 4 IQR (3–4.75)	NA	Median: 5 (IQR: 4, 5)	NA	NA	NA	Median: 6.5 (IQR: 2, 2.75)	
Cooper^13^	DC	NA	Median: 3 (IQR 2–5)	NA	14.4 ± 6.8	14 (19)	Median: 28 (IQR: 21, 62)	Median: 13 (IQR: 10, 18)	27
	Medical therapy	NA	Median: 4 (IQR 3–5)	NA	19.1 ± 8.9	15 (18)	Median: 37 (IQR: 24, 44)	Median: 18 (IQR: 13, 24)	14
Nirula^9^	DC	NA	NA	NA	11.7 ± 11.8	63 (30)	16.4	10.9	NA
	Medical therapy	NA	NA	NA	12.3 ± 13.1	59 (28)	13.7	8.5	
Hutchinson^11^	DC	NA	Favorable: 86 (43%); Unfavorable: 115 (57%)	Favorable: 88 (45%); Unfavorable: 106 (55%)	Median: 14.5 (IQR: 1.7, 18)	54 (26.8)	NA	Median: 15.0	33
	Medical therapy	NA	Favorable: 65 (35%); Unfavorable: 123 (65%)	Favorable: 58 (32%); Unfavorable: 121 (68%)	Median: 17.1 (IQR: 4.2, 21.8)	92 (48.9)	NA	Median: 20.8	18

DC, Decompressive Craniectomy; ICP, Intracranial Pressure; ICU, intensive care unit; IQR, Interquartile Range; GOS, Glasgow Outcome Scale; LOH, Length of Hospitalization; LO-ICU, Length of ICU Stay; NA, Not Available.

### Overall mortality

**Figure 2:**
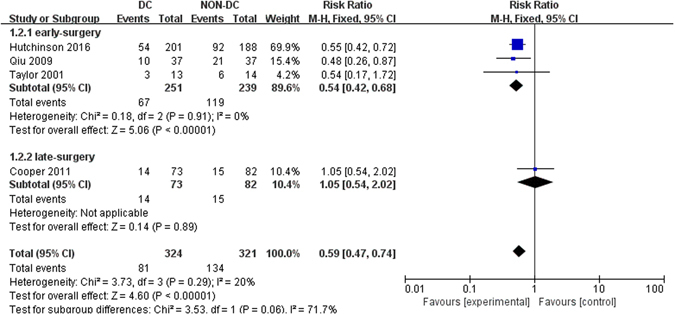
Forest plots for the effect of DC versus NON-DC on overall mortality. DC, Decompressive Craniectomy.

Four RCTs were included to quantitatively evaluate the effect of DC on overall mortality after traumatic ICH^6, 11, 13, 14^. In view of no significant heterogeneity among studies (Q = 3.73, *P* = 0.29, *I* ^2^ = 20%), we used fixed-effects model in the analysis. The *P* value had statistical significance [Risk Ratio (RR), 0.59; 95% CI, 0.47–0.74, Z = 4.60, *P* < 0.001], which indicated that patients in DC group had half the risk of death as compared with those in medical care group (Fig. 2). The statistical significance was stable in the subgroup of early-surgery group (*P* < 0.001) with little evidence of heterogeneity (*I* ^2^ = 0). We identified no difference in the subgroup of late-surgery group (*P* = 0.89) (Fig. 2).

Six more observational studies^9, 15–19^ explored the effect of DC on mortality rate in patients with traumatic ICH. Four of them^15, 16, 18, 19^ reported reduced mortality rate for patients undergoing DC compared with Non-DC treatment, whereas one study^9^ detected similar mortality and another one^17^ had incomplete data.

### Glasgow Outcome Scale (GOS) and extended Glasgow Outcome Scale (GOS-E)

**Figure 3:**
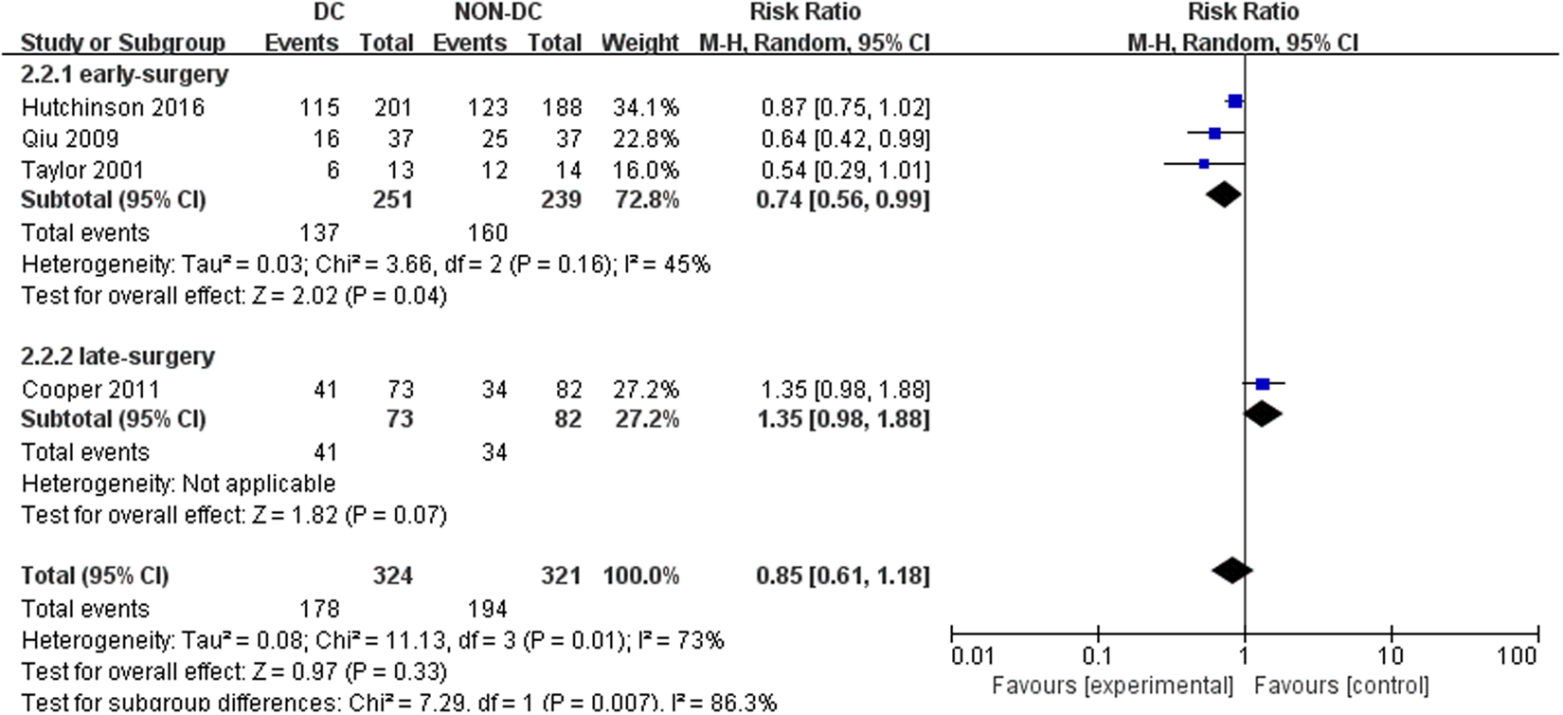
Forest plots for the effect of DC versus NON-DC on GOS scores at 6 months. DC, Decompressive Craniectomy; GOS, Glasgow Outcome Scale.

When analyzed as dichotomous data, GOS/GOS-E scores at six months from four RCTs were pooled for the effect of DC on functional outcomes and GOS/GOS-E scores of no less than four were considered as favorable^6, 11, 13, 14^. According to the summary results, no significant difference was found between two groups (RR, 0.85; 95% CI, 0.61–1.18, Z = 0.97, *P* = 0.33, Fig. 3). However, in the subgroup of early surgery, it seemed that DC could improve patients’ functional outcomes compared with patients without DC (RR, 0.74; 95% CI, 0.56–0.99, Z = 2.02, *P* = 0.04, Fig. 3). There was no statistically significant result in the subgroup of late-surgery (*P* = 0.07). What’s more, despite of a neutral effect on 6-month GOS-E scores between two groups, improved prognosis in RESCUEicp trial based on 12-month GOS-E after DC was presented, which suggested potential benefit of DC under long-term follow-up.

When analyzed as continuous data, two studies^11, 13^ were availabale and mean GOS-E scores in Cooper *et al*.^13^ were 3.41 ± 1.76 (mean ± standard deviations (SD), DC group) and 4.05 ± 1.96 (Non-DC group), which were 3.31 ± 1.90 (DC group) and 2.88 ± 2.18 (Non-DC group) in Hutchinson *et al*.^11^. However, owing to the high heterogeneity betewen the two studies (*I* ^2^ = 88%), we chose to narratively describe the results instead of pooling them. In Cooper *et al*.^13^, DC was associated with worse GOS-E scores (*P* = 0.03) and more unfavorable outcomes compared with medical care, while in Hutchinson *et al*.^11^, DC was related to better GOS-E scores but similar unfavorable outcomes (*P* = 0.12). In view of the discrepancies, more large scale RCTs were needed to unravel the effect of DC on functional outcomes.

Five more observational studies^15–19^ assessed the effect of DC on GOS score in patients with traumatic ICH. Improved outcome in DC group was detected in two studies^16, 18^ in comparison with medical care, with similar outcome in two studies^15, 17^ and worse outcome in one study^19^.

### ICP reduction

**Figure 4:**
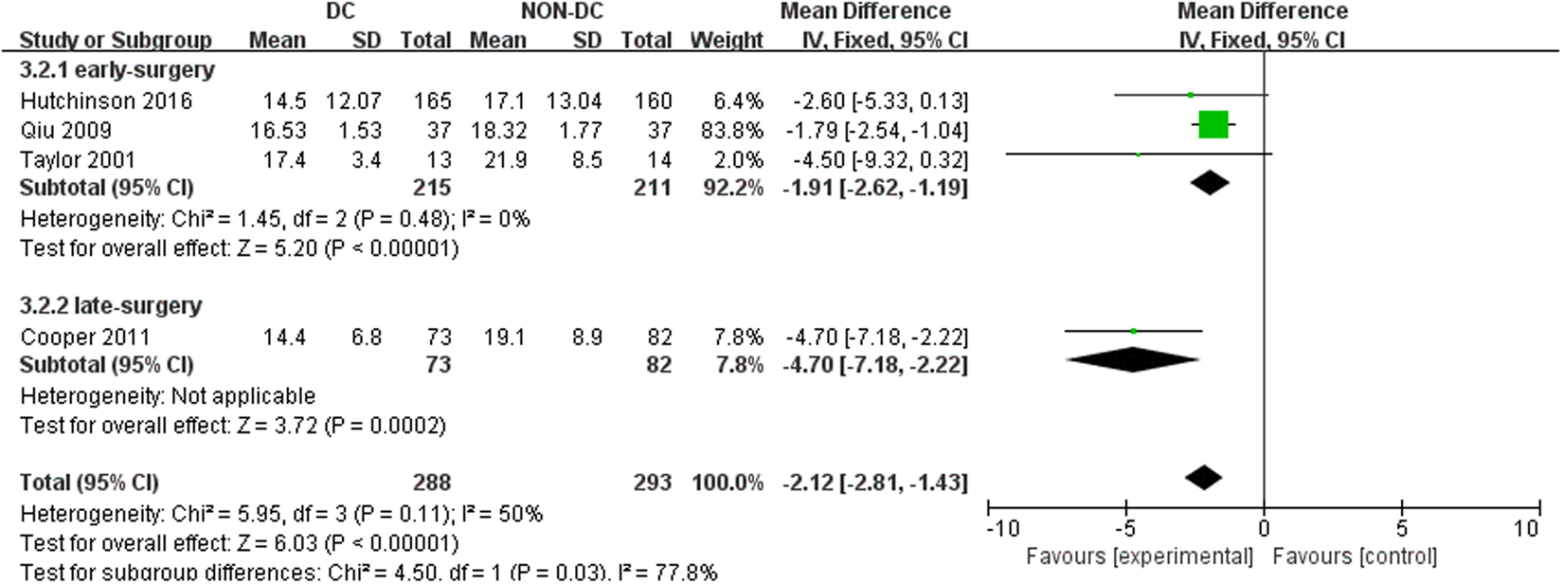
Forest plots for the effect of DC versus NON-DC on ICP reduction. DC, Decompressive Craniectomy; ICP, Intracranial Pressure.

Four studies were available in quantitatively assessing the effect of DC on ICP levels^6, 11, 13, 14^. The data were pooled using fixed effects model and the *P* value was statistically significant [mean difference (MD), −2.12 mm Hg; 95% CI, −2.81 to −1.43, Z = 6.03, *P* < 0.001] with no significant heterogeneity (Q = 5.95, *P* = 0.11, *I* ^2^ = 50%). The results demonstrated that there was a significant reduction of ICP in patients receiving DC as compared with those receiving medical care. The statistical significance was stable in both subgroups (*P* < 0.001 for early-surgery and *P* = 0.0002 for late-surgery) (Fig. 4).

ICP was reported as outcomes in three more observational studies^15, 17, 19^ with all of them favoring effective control of ICP under DC.

### Length of hospitalization (LOH) and Length of intensive care unit (ICU) stay (LO-ICU)

**Figure 5:**
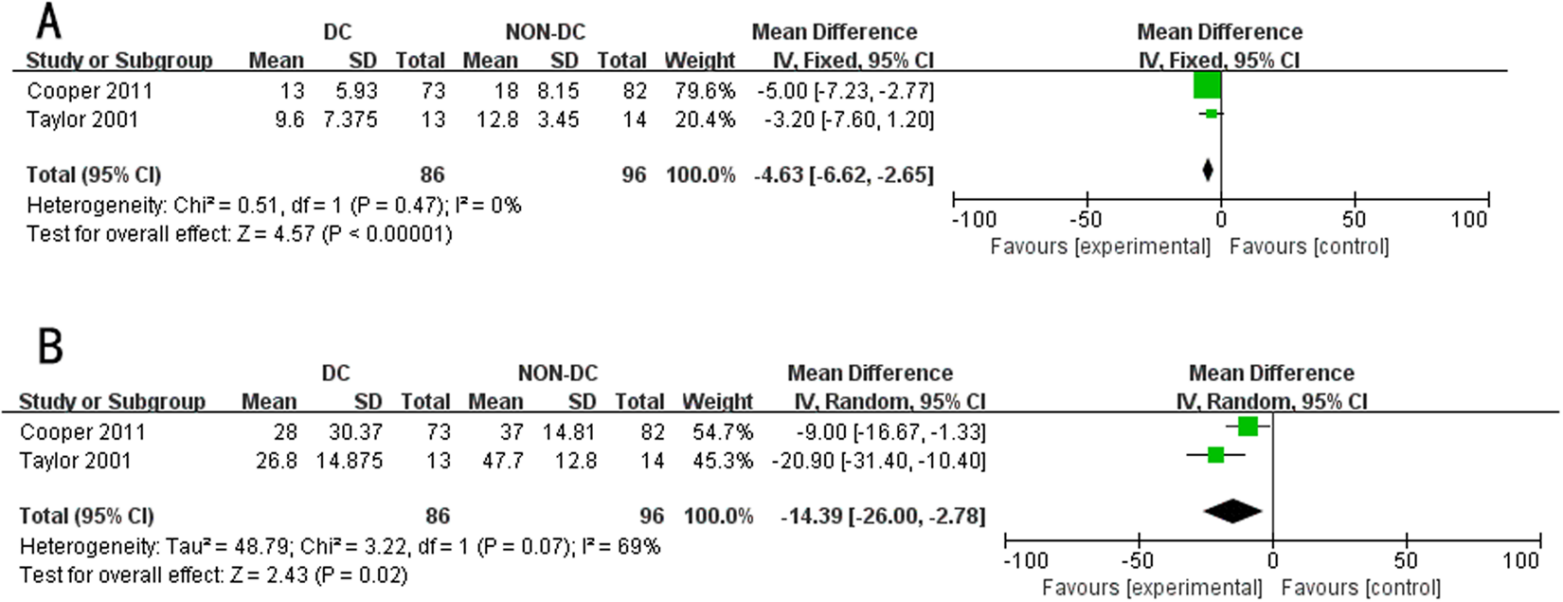
Forest plots for the effect of DC versus NON-DC on length of ICU and hospital stay. (**A**) length of ICU stay; (**B**) Length of hospital stay. DC, Decompressive Craniectomy; ICU, intensive care unit.

Length of ICU stay and hospital stay could be extracted from two RCTs involving 182 patients^13, 14^. Findings from quantitatively analysis suggested that the ICU stay in the DC group was about five days less than that in the non-DC group (MD, −4.63 days; 95% CI, −6.62 to −2.65, Z = 4.57, *P* < 0.001, Fig. 5A), and the hospital stay in the DC group was about 14 days less when compared with non-DC group (MD, −14.39 days; 95% CI, −26.00 to −2.78, Z = 2.43, *P* = 0.02, Fig. 5B). Two more observational studies^9, 17^ were available in the analysis of LOH and LO-ICU with one of them^9^ detecting prolonged LOH and LO-ICU in DC group and another one study^17^ favoring prolonged LO-ICU in DC group, which were different from results of quantitative synthesis.

### Complications

**Figure 6:**
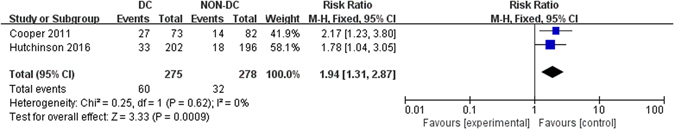
Forest plots for the effect of DC versus NON-DC on complications. DC, Decompressive Craniectomy.

Two RCTs containing 553 patients assessed the incidence of complications after intervention^11, 13^. There was significant difference between DC and non-DC group with pooled RR of 1.94 [95% confidence interval (CI), 1.31–2.87, Z = 3.33, P = 0.0009, Fig. 6] and no heterogeneity (*I* ^2^ = 0), which suggested the incidence of complications was higher in patients undergoing DC than those undergoing traditional medical treatment. One more observational study^9^ compared the incidence of complications after DC and medical care and reported increased incidence of complications after DC.

### Sensitivity Analyses

We performed sensitivity analyses for overall mortality, GOS scores and ICP reduction. In sensitivity analysis for mortality, similar results were detected when removing the study by Cooper *et al*. (*P* < 0.01)^13^, Qiu *et al*. (*P* < 0.01)^6^ or Taylor *et al*. (*P* < 0.01)^14^. Whereas pooled results turned to be non-significant when removing Hutchinson *et al*. (*P* = 0.07)^11^. For GOS at six months, no change was found until excluding the study by Cooper *et al*. (*P* = 0.04)^13^. For ICP level, there was no change when excluding studies one by one.

## Discussion

ICH after TBI was related to the increased incidence of mortality and morbidity in most studies^1, 20^, and DC was said to be effective in lowering ICP and improving outcomes in ischemic and traumatic injury^6, 21^. The present systematic review and meta-analysis confirmed that DC could significantly lower ICP, reduce mortality rate, but was correlated to an increased incidence of complications. Quantitative results of decrease of LOH and LO-ICU could not be supported by observational studies. While DC was associated with similar risk of favorable outcome at six months compared with traditional management, early surgery (time interval to surgery <36 h) resulted in improved outcomes in subgroup analysis for GOS score at six months.

Three studies^14, 16, 17^ focused on children, with the remaining seven focusing on adults. Besides studies with incomplete data, DC could significantly reduce mortality and ICP in children^14, 16^, while its benefit on functional outcomes can only be found in two studies^14, 16^ with another one^17^ favoring similar effect of DC and conservative treatment. As for the LOH and LO-ICU, data was limited to indicate a significant effect of DC in children. Generally, our findings on mortality, ICP and GOS apply to adults and children as well.

After TBI, mass effect caused by brain swelling and intracranial hematomas would lead to the elevation of ICP, which might decrease the cerebral perfusion pressure (CPP) and then bring about brain ischemia^2, 22^. Theoretically, DC could lower ICP by allowing the expansion of swollen brain and then increase cerebral blood flow (CBF), resulting in reduced damage size and improved outcome^12^. Some previous studies have also confirmed the effect of DC on CBF and outcome of patients with ICH^6, 14, 23^. Therefore, despite lacking of level I evidence, DC was routinely used in the management of ICH in some trauma centers. However, overall opinion on the effect of DC on patients with traumatic ICH was inconsistent and some authors found that DC might even lead to worse outcomes than traditional therapies^13, 19^. Most early researches were retrospective and it was not until this decade that a few RCTs emerged to unravel the issue.

The first RCT was published in 2001, which randomly assigned 27 children with RICH after TBI into standardized management alone or standardized management plus DC^14^. Despite of the small sample size, the trial detected that children treated with standardized management plus DC had lower ICP (17.4 ± 3.4 mm Hg versus 21.9 ± 8.5 mm Hg), fewer episodes of ICP > 20 mm Hg (107 versus 223) and better functional outcome (54% versus 14%) compared with those treated with standardized management alone. In another RCT, 74 patients with brain swelling were randomly divided into unilateral DC group and unilateral routine temporoparietal craniectomy group^6^. Decreased ICP (72 h after injury, 15.98 ± 2.24 mm Hg versus 21.05 ± 2.23 mm Hg), reduced mortality rate (27% versus 57%) and improved neurological outcomes (56.8% versus 32.4%) in patients receiving DC were suggested in the findings. The third RCT, which was the first large scale RCT (DECRA), randomly assigned 155 adults with TBI and RICH to receive bifrontotemporoparietal DC or standard care^13^. Patients in DC group had shorter duration of ICH (ICP > 20 mm Hg), fewer days in ICU (*P* < 0.001) and greater risk of an unfavorable outcome [Odd Ratio (OR), 2.21; 95% CI, 1.14 to 4.26; *P* = 0.02] than those in standard care group, whereas the mortality rate at six months was similar in two groups. This trial was criticized for the fact that the recruitment criterion of ICP > 20 mm Hg for 15 minutes did not necessarily indicate an ongoing secondary brain injury and any potential benefit derived from DC might be offset by surgical morbidity. The latest RCT, RESCUEicp trial, was designed to assess the effect of DC as a last-tier therapy in patients with TBI and RICH (ICP > 25 mm Hg for 1 to 12 hours)^11^. RESCUEicp was the largest RCT so far, in which 408 patients with TBI and RICH were randomized to undergo DC or medical care. The findings revealed that DC contributed to lower ICP and mortality rate, higher incidence of vegetative state, lower severe disability, and upper severe disability as compared with medical care at six months. Despite similar risk of favorable outcomes in two groups (*P* = 0.12), patient in DC group had better functional outcomes than those in control group at 12 months (*P* = 0.01). In view of defects in the design of DECRA trial and results in subgroup analysis for GOS score at six months (*P* = 0.04), we suspected possible benefits of DC on long-term functional outcomes, which was to be confirmed in further large scale RCTs.

Several systematic review and meta-analysis are available exploring the effect of DC on patients with traumatic ICH. A Cochrane review published in 2006 only included one RCT and found little evidence to support the routine use of secondary DC^4^. A meta-analysis in 2012 examined the contribution of DC in reducing ICP and increasing CPP in patients with TBI and RICH^24^. They found that DC could effectively lower ICP and raise CPP, but they did not analyze the role of DC in functional outcomes and mortality rate. A recent meta-analysis based on three RCTs which had different results to our study reported that DC, when compared with conventional treatment, could reduce ICP and decrease hospital stay, but was associated with similar mortality rate^12^. Results for functional outcomes were not discussed in the article. Our study has advantages in including the latest RCT with the largest sample size and acceptable recruitment criterion, which account for the maximum weight in all analysis in the current study. Moreover, we conducted quantitative synthesis for the functional outcomes and complications rate after interventions for the first time.

There are several limitations in our study. Firstly, different biases exist due to the defects of meta-analysis itself, such as selection bias and publication bias. Patients receiving DC might have a higher preoperative ICP than those receiving traditional therapies and tend to have a worse outcome^12^. The language was limited to English, which might lead to the overlook of non-English studies. Secondly, heterogeneity among studies was significant in present research, which might come from discrepancies in the timing, type and technique of operation, patients’ age and baseline conditions of TBI^6^. Therefore, caution was needed in interpretating these results. Thirdly, the number of pertinent high-quality trials was limited. Only one large scale RCT with acceptable inclusion criterion was available^11^. Fourthly, although we did quantitative synthesis for the overall incidence rate of complications, pooled analysis for each detailed complications of DC were not conducted in our study owing to the lack of complete data. This may result in some misconception. For example, DC could decrease the incidence of cenencephalocele, despite an elevation was found in the incidence of other complications like subdural effusion, intracranial hematoma and hydrocephalus^6^. Finally, although ICP was routinely monitored in the management of TBI patients, its prognostic relevance is limited compared with CBF and oxygenation, which has proved to be intimately related to neurological outcomes after TBI^19, 25–27^. However, CBF and metabolism are seldomly evaluated in common practice due to the inconvenience, expensiveness and exposure to radiation. Moreover, despite of the significant effects on controlling ICP levels and maintaining CBF, DC might lead to significantly lower cerebral metabolic rate of oxygen compared with medical management, which may account for the non-significant improvement of functional outcomes after DC^19^. Previous studies suggested DC failed to respond to the mitochondrial damage, resulting in cellular energy crisis and edema and eventually the poor prognosis^19, 25, 28^.

## Conclusions

Despite the limitations, our findings presented certain clinical implications that DC seemed to effectively lower ICP, reduce mortality rate but increase incidence of complications, meanwhile its benefit on functional outcomes was not statistically significant. More large scale RCTs with long-term follow-up were needed to confirm the potential benefit of early surgery on functional outcomes and the exact effect of DC on LOH and LO-ICU after traumatic ICH. Caution was required when interpreting these results due to the limited number of large scale RCTs and significant heterogeneities among included studies.

## Materials and Methods

### Search Strategy and Selection Criteria

Our systematic review and meta-analysis was performed following the guidelines of Preferred Reporting Items for Systematic Reviews and Meta-Analysis: The PRISMA Statement^29^. We conducted a comprehensive search of the medical literature using PubMed (inception to October 2016), EMBASE (inception to October 2016), Cochrane Controlled Trials Register (October 2016), Web of Science (inception to October 2016) and http://clinicaltrials.gov/ on October 31th 2016. Search terms were (traumatic brain injury) AND (intracranial hypertension OR high intracranial pressure OR elevated intracranial pressure) AND (craniectomy). The reference lists of the original studies were also examined. We restricted the language of publications to English.

Two authors (D. F. Z., Q. X.) screened the titles and abstracts independently and then potentially eligible studies were assessed by reading full text. Studies were included in our review if they: 1) were RCTs or 2-arm studies (quantitative synthesis were performed for RCTs only); 2) recruited patients suffering TBI and receiving DC as an intervention. We excluded studies if they: 1) recruited patients with spinal cord injury or mass lesions; 2) did not report quantitative outcome data. Disagreements were consulted by joint review.

### Data Extraction

All data were extracted by two authors (J. G. C., Y. D.) independently and then checked by a third reviewer (L. J. H.). The following data were extracted for each study: first author; study design; publication year; number of patients in each group; patients’ gender and age; the proportion of male; severity of patients’ disease; time interval to the treatment; detailed description of treatment; ICP levels before and after intervention; overall mortality; LOH; LO-ICU; GOS score at three, six, twelve mouths and complications.

### Outcomes

Primary outcome was mortality at six months after randomization. Secondary outcomes included functional outcome at six months, ICP level, LOH an LO-ICU, complications. GOS scores of one to five represent death, vegetative state, severe disability, moderate disability, good recovery, respectively^30^. GOS-E scores of one to eight represent death, vegetative state, lower severe disability, upper severe disability, lower moderate disability, upper moderate disability, lower good recovery, and upper good recovery, respectively^11^. Unfavorable outcomes were defined as GOS/GOS-E score of one to three at six months. ICP was the pressure inside the brain tissue and CSF with normal range of 7–15 mmHg and traumatic ICH due to mass effect or brain edema may be fatal^31^.

### Data Analysis

A systematic descriptive review was conducted on all included studies. For RCTs, we calculated the *I* ^2^ statistic and Chi-square test to assess the homogeneity among studies. Significant homogeneities among studies were suggested and random-effects model was used in the synthesis if *I* ^2^ exceeded 50% and the *P* value was less than 0.10. Otherwise, we used fixed effects model. Dichotomous data such as the overall mortality were combined using RR, while continuous data, such as ICP, LOH and LO-ICU stay, were combined using MD. GOS score was analyzed as both dichotomous and continuous variable as well, and only studies with sample sizes of more than 60 in each group were included when it was analyzed as continuous measures due to its trend of skew distribution. Means and SDs were calculated with Microsoft Office Excel 2007 (Microsoft Corporation, Washington) if the distribution of participants was available. According to the Cochrane handbook, median was estimated to be mean and SD was calculated as width of IQR divided by 1.35^32^. In the quantitative synthesis, *P* < 0.05 was considered as statistically significant. We performed a subgroup analysis according to the timing of DC. Studies were divided into early-surgery and late-surgery group with the threshold defined by time interval to DC of 36 hours after injury. Sensitivity analyses were performed by excluding one study at a time to test the stabilization of our results. Publication bias were not assessed because of the limited studies in the review. Statistical analyses were conducted with Review Manager (RevMan), version 5.3 (Copenhagen: The Nordic Cochrane Centre, The Cochrane Collaboration, 2014).

### Quality Assessment

The quality assessment was performed independently by two review authors (Y. J., J. Y. W.), with discrepancies resolved by discussion. We assessed the quality of RCTs based on the quality domains in the Cochrane risk of bias tool: random sequence generation, allocation concealment, blinding of participants and personnel, blinding of outcome assessment, incomplete outcome data, selective reporting and any other potential bias. Each domain was rated as high, low or unclear.

### Ethic Review

Meta-analysis does not require Institutional Review Board (IRB) review.

## Electronic supplementary material

Supplementary Information
