# Supplementary material for: Decompressive craniectomy in the management of intracranial hypertension after traumatic brain injury: a systematic review and meta-analysis
Source: Sci Rep. 2017 Aug 18;7:8800. doi: 10.1038/s41598-017-08959-y (PMC5562822; doi:10.1038/s41598-017-08959-y)

Title: Decompressive craniectomy in the management of intracranial hypertension after traumatic brain injury: a systematic review and meta-analysis.

Author list

Danfeng Zhang[1], Qiang Xue[1], Jigang Chen[1], Yan Dong, Lijun Hou, Ying Jiang*, Junyu Wang*

Supplementary Figure 1: Risk of bias for RCTs

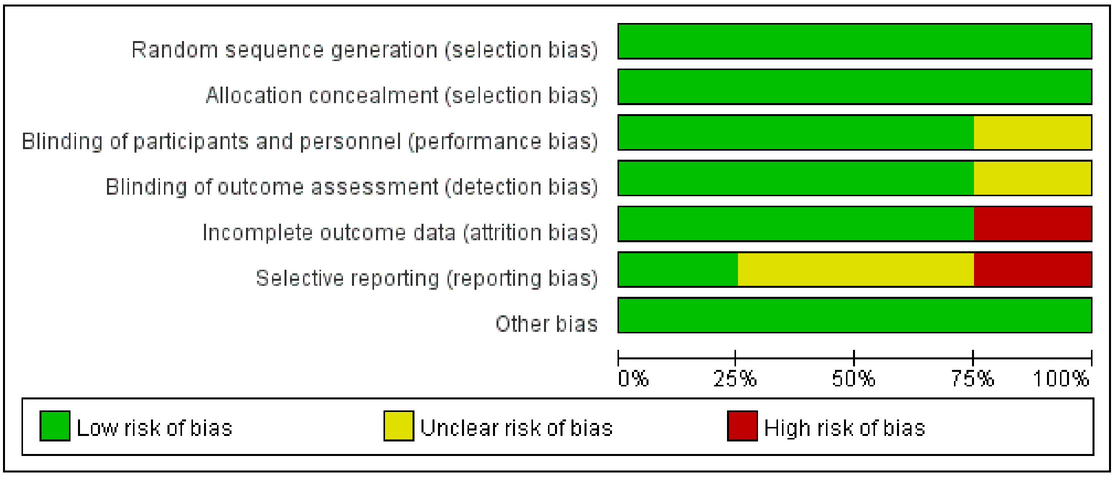

Supplementary Figure 2: Risk of bias summary for RCTs.

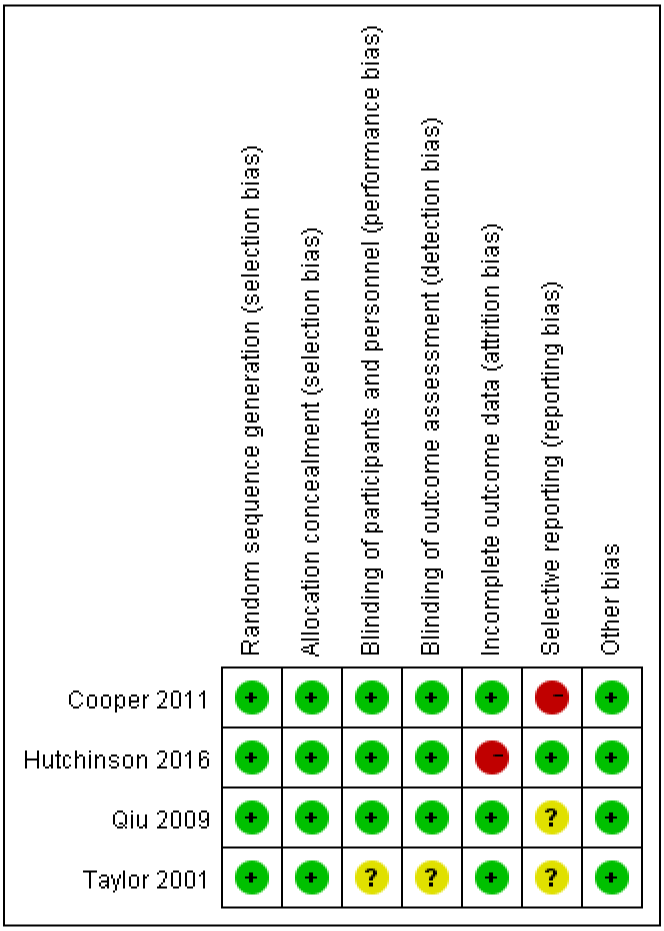

Supplement: Supplementary file 1 — Supplementary Information [file 41598_2017_8959_MOESM1_ESM.pdf]
